# Molecular Model for Linear Viscoelastic Properties of Entangled Polymer Networks

**DOI:** 10.1021/acs.macromol.4c01429

**Published:** 2024-10-15

**Authors:** Andrei A. Gusev, Tim Bernhard

**Affiliations:** Department of Materials, ETH Zürich, CH-8093 Zürich, Switzerland

## Abstract

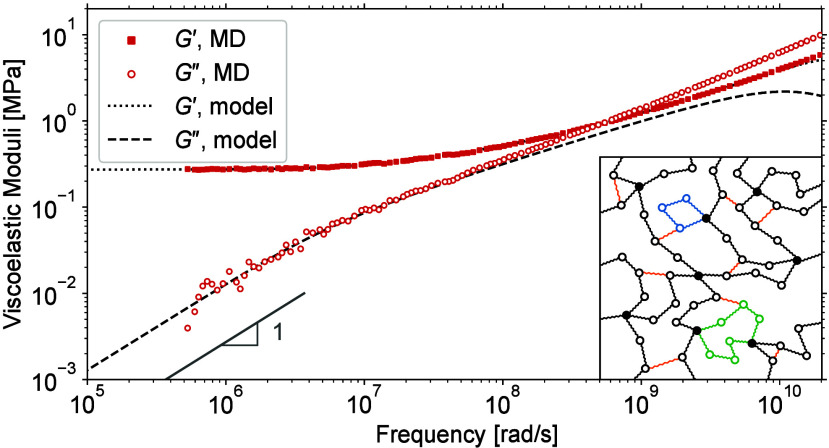

A molecular Kuhn-scale model is presented for the stress relaxation dynamics of entangled polymer networks. The governing equation of the model is given by the general form of the linearized Langevin equation. Based on the fluctuation–dissipation theorem, the stress relaxation modulus is derived using the normal mode representation. The entanglements are introduced as additional entropic springs connecting internal beads of the network strands. The validity of the model is assessed by comparing predicted stress relaxation modulus and viscoelastic storage and loss moduli with the estimates from molecular dynamics (MD) simulations, using the same computer models. A finite element procedure is proposed and used to assemble the network connectivity matrix, and its numerically solved eigenvalues are used to predict the linear stress relaxation dynamics. Both perfect (fully polymerized stoichiometric) and imperfect networks with different soluble and dangling structures and loops are studied using mapped Kuhn-scale network models with up to several dozen thousand Kuhn segments. It is shown that for the overlapping ranges of times and frequencies, the model predictions and MD estimates agree well.

## Introduction

The Rouse model was the first successful molecular theory of polymer dynamics.^1−4^ Unfortunately, in the dilute solutions its predictions do not agree with experiments. For example, in the Rouse model the diffusion coefficient of the center of mass and the rotational relaxation time depend on the polymer molecular weight *M* as *D*_G_ ∝ *M*^–1^ and τ_r_ ∝ *M*^2^, respectively, whereas in experiments *D*_G_ ∝ *M*^–ν^ and τ_r_ ∝ *M*^3ν^, where in a θ-solvent the exponent ν = 1/2 while in a good solvent ν ≃ 3/5. The main reason for this discrepancy is the neglect of the hydrodynamic interactions that couple the motion of the surrounding beads in solutions, which was later rectified in the Zimm model of polymer dynamics.^2−5^ However, the Rouse model turned out to be useful in describing the dynamics of short unentangled polymer chains in melts where the hydrodynamic interactions are screened by the presence of other chains.^2−4^ For melts of entangled chains the Rouse model is also helpful in describing the dynamics at short times before the entanglement effects become dominant at longer times, which is theoretically described by the tube models of entangled polymer dynamics.^2−4,6,7^

The linear viscoelastic properties of polymer melts, networks and gels are encoded in the stress relaxation modulus *G*(*t*), which can be used to relate different viscoelastic experiments using the Boltzmann superposition principle. In molecular simulations, the *G*(*t*) can be extracted from equilibrium stress fluctuations using the Green–Kubo formalism,^8^ and it has been confirmed by simulations that the Rouse model is indeed helpful for studying and understanding the dynamics of melts of unentangled chains and the short-time dynamics of melts of entangled chains.^9−11^

Chompff et al.^12,13^ extended the Rouse model to entangled polymer networks and as an application considered model mesh-like networks with different loops. The entanglements were accounted for by an additional frictional force due to the velocity difference between the beads at the entanglement point. Following on from the Graessley’s works,^14,15^ Kloczkowski et al.^16,17^ studied various model tree-like phantom networks and derived their relaxation spectra. Recently, a modified sticky Rouse model has been proposed and used for associative polymers and networks.^18,19^ Assuming model arrangements of classical affine and phantom network model, the relaxation spectra have been derived for both permanent and transient dual polymer networks using the graph theory. Vandoolaeghe and Terentjev^20^ merged the Rouse model with the tube-model description of the network elasticity proposed by Edwards.^21,22^ Using a dynamic stress tensor approach, both equilibrium and linear viscoelastic responses of entangled polymer networks were investigated assuming analytically tractable model arrangements.

In the molecular simulations with hard-core repulsive potentials, such as for example atomistic and coarse-grained Kremer-Grest molecular dynamics (MD) simulations, the entanglements are accounted for automatically, but relatively short integration time steps are required to secure that the polymer chains do not cross each other and themselves. As a consequence, such hard-core simulations are typically limited to relatively small systems and short times. Coarse-grained soft-core molecular simulations allow for more efficient studies; however, they require additional slip-links or slip-springs to account for the chain uncrossability.^23−34^ The slip-springs are commonly added on the smallest length scale resolved by a particular simulation method and various mechanisms of their creation, migration and destruction have been proposed and implemented in different coarse-grained simulation methods such as dissipative particle dynamics (DPD), Brownian dynamics and hybrid particle field methods, and their hierarchical multiscale combinations.^26,29,31−34^ Most of the soft-core simulations have been devoted to polymer melts. As for the polymer networks, Megariotis et al.^35^ used a slip-spring Brownian dynamics method to study the equilibrium and linear relaxation moduli of model polymer networks. Recently, Masubushi and Uneyama^36^ used a slip-spring model to study the gelation process of entangled polymer melts. Following on from a slip-spring DPD model developed for polymer melts by Langeloth et al.,^29^ a modified model has been used by Schneider et al.^37^ to study the viscoelastic properties of vulcanized polyisoprene rubbers, and the results were compared with both experimental data and literature Kremer-Grest MD simulations of Gula et al.^38^ Recently, Schneider and de Pablo used slip-spring DPD simulations to study the dynamics of entangled polymers at interfaces between liquids, liquids and vapor, and liquids and solids.^39^

In this work, a molecular theory for the linear stress relaxation dynamics of entangled polymer networks is presented. The theory does not rely on any simplified, analytically tractable model network arrangements but instead directly works with representative periodic network models consisting of several dozen thousand and more Kuhn segments. For the first time as we know, the relaxation dynamics of entangled polymer networks is theoretically derived using a general Langevin formalism. A finite element procedure is proposed and used to assemble the network connectivity matrix, and its numerically solved eigenvalues are employed to predict the linear stress relaxation dynamics. As in the literature slip-spring simulations, the entanglements are introduced on the smallest resolved length scale by means of additional Kuhn segments connecting nearby beads. Molecular dynamic simulations are employed to obtain estimates of the stress relaxation modulus and viscoelastic storage and loss moduli of both perfect and imperfect end-linked polymer networks with different soluble and dangling structures and loops, and the estimates are used to assess the scope of validity of the presented model using mapped Kuhn-scale representations of the MD network models.

## Results and Discussion

### Molecular model of Network Dynamics

**Figure 1:**
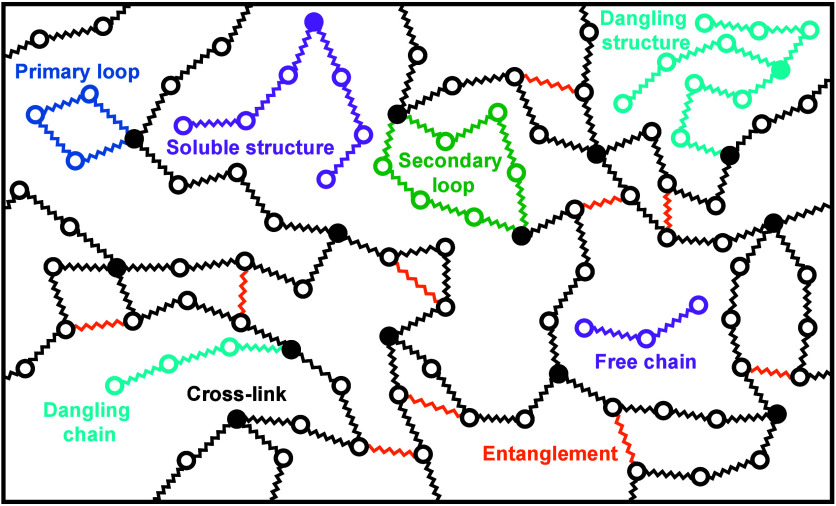
A model of an end-linked polymer network. Tetra-functional cross-linkers (filled circles) are end-linked by identical difunctional precursor chains consisting of three beads connected by two springs (free chain). Upon curing, each terminal precursor bead can react with an unreacted site of a cross-linker. The entanglements are modeled by single springs connecting two neighboring nonbonded internal beads.

Consider a Gaussian polymer network consisting of *N* beads linked by harmonic entropic springs of natural length 0 and spring constant *k*, see Figure 1.

The governing equation of the model is given by the general form of the linearized Langevin equation:^2,3^
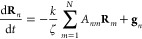
1where vectors **R**_*n*_ are the positions of the beads, *t* is time, ζ and *k* are the friction coefficient and the spring constant, respectively, **A** is a constant integer symmetric connectivity matrix representing pairwise interactions among the beads connected by the springs, and **g**_*n*_ is a random force with the mean and variance given by
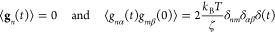
2where *k*_B_ and *T* are the Boltzmann constant and the temperature, respectively, and ⟨···⟩ denotes the ensemble average.

The normal modes **X**_*p*_ are defined as
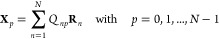
3where the columns of the orthonormal matrix **Q** are unit eigenvectors of the matrix **A**. The equation of motion for **X**_*p*_ has the following form:
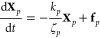
4where ζ_*p*_, *k*_*p*_ and **f**_*p*_ are the friction coefficient, spring constant and random force of the normal mode *p*, respectively.

Using eqs 1 and 3 one obtains:
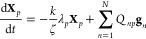
5where λ_*p*_ are the eigenvalues of matrix **A**. Comparing eqs 4 and 5 one finds
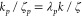
6Using eqs 2, 4, and 5 and the relation **Q**^T^**Q** = **I**, where **I** is the identity matrix, one can readily show that the force **f**_*p*_ has the same mean and variance as the force **g**_*n*_ of eq 2.

Since matrix **A** is integer symmetric, the eigenvalues λ_*p*_ are real and nonnegative. The normal modes with λ_*p*_ = 0 correspond to the translational motion while those with λ_*p*_ > 0 to the vibrational motion of independent dumbbell molecules with modal relaxation times
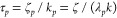
7Notice that the relaxation time τ_*p*_ is determined by the ratio of ζ_*p*_ and *k*_*p*_. Therefore, the choice of either ζ_*p*_ or *k*_*p*_ is arbitrary, and we choose a pair of them as follows:
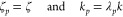
8

The time correlation functions of the normal modes can be written at once by analogy with the known expressions for a free Brownian particle and a harmonic oscillator:^2,3^
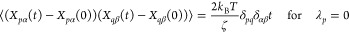
9
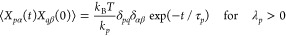
10where δ_*pq*_ and δ_*αβ*_ are components of the Kronecker delta.

In terms of the normal modes, the relaxation shear stress *δτ* can be written as follows:
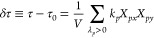
11where τ and τ_0_ are the total and residual stress, respectively, *V* is the volume occupied by the network, and the summation is taken over all vibrational modes. On the basis of the fluctuation–dissipation theorem,^8^ using eqs 10 and 11 and noticing that the motions in the *x*- and *y*-directions are independent, the stress autocorrelation function *C*(*t*) is calculated as follows:
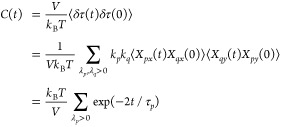
12The stress relaxation modulus *G*(*t*) is given by
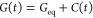
13where *G*_eq_ is the equilibrium shear modulus, which is unrelated to thermal stress fluctuations and cannot be obtained from them.^40−42^ However, for end-linked polymer networks formed from bulk, there exists an accurate theoretical solution for *G*_eq_ given by the Miller-Macosko theory (MMT).^43−45^

Eqs 12 and 13 provide a solution for the stress relaxation modulus *G*(*t*) of an arbitrary Gaussian polymer network. The solution automatically accounts for the presence of various topological network defects such as different soluble and dangling structures and loops, see Figure 1. The solution is also valid for melts of Gaussian chains, where it reduces to the classical solution obtained by P. E. Rouse in 1953.^1^

### Rouse Model

**Figure 2:**
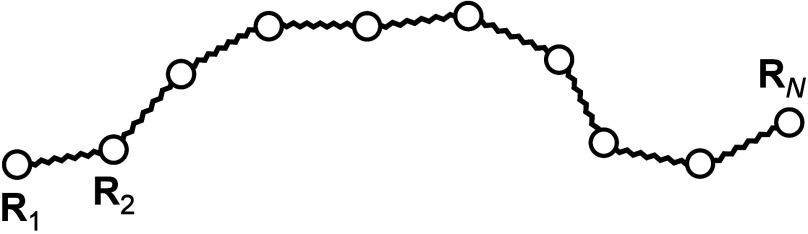
A Rouse model.

Consider a single Gaussian chain consisting of *N* beads linked by harmonic entropic springs of natural length 0 and spring constant *k*, see Figure 2.

The governing equations of this model can be written as follows:
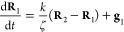
14
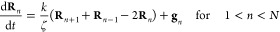
15
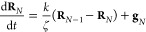
16where **R**_*n*_ is the position vector of bead *n*. The connectivity matrix **A** is as follows:
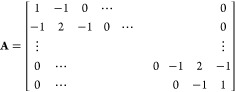
17The eigenvalues of matrix **A** are given by^46^
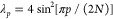
18which are nonnegative and in ascending order, with λ_0_ = 0 related to the translational motion of the center of mass of the molecule and λ_*p*_ > 0 to the vibrational motion of (*N*–1) independent dumbbells.

For melts, the equilibrium modulus *G*_eq_ = 0 and using eqs 12 and 13 one obtains:
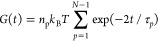
19where *n*_p_ is the number density of polymer molecules.

One commonly uses a continuous elastic-thread approximation to formulate the Rouse model.^2−4^ Accordingly, one assumes that the beads are continuously distributed along the polymer chain and, by assuming *p* ≪ *N*, approximates eq 18 as
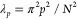
20Then it follows that for the dumbbell modes
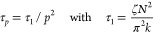
21and hence
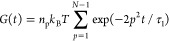
22which is a traditional literature expression for the stress relaxation modulus of the Rouse model in the continuous approximation.^2−4^

For *t* ≪ τ_1_, the sum over *p* in eq 22 can be approximated by an integral over *p*:
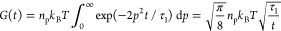
23Therefore, *G*(*t*) decays as a power law *t*^–1/2^ on the intermediate times τ_*N*–1_ ≪ *t* ≪ τ_1_. Noticing that τ_1_ scales as *N*^2^ by eq 21 and that due to the density constraint, the number density of polymer molecules *n*_p_ is proportional to 1/*N*, one concludes that on the intermediate times *G*(*t*) is independent of *N*.

### Entanglements

As mentioned in the Introduction, in the contemporary soft-core molecular simulations the entanglements are usually represented by means of the slip-springs added on the smallest length scale resolved by the simulation method. Following from this, we introduce the entanglements as additional entropic springs connecting adjacent beads of the network strands, see Figure 1. These springs alter both the structure of the connectivity matrix **A** and the resulting spectrum of the normal modes but change neither the dimension of matrix **A** nor the number of the normal modes. The entanglement density is a free system-specific parameter, and for the end-linked networks studied below we shall use the Miller-Macosko theory (MMT) to define the entanglement density based on the known entanglement modulus of fully polymerized networks, see eq 26.

### Assembly procedure

The Rouse connectivity matrix **A** of eq 17 can be assembled by realizing that the contribution of a spring to **A** is given by a reduced stiffness (Hessian) matrix:
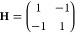
24This idea can be readily used to assemble the connectivity matrix **A** of Gaussian networks of arbitrary topology, including those under periodic boundary conditions: Upon assembly, a spring connecting beads *n* and *m* contributes 1 to the diagonal entries *nn* and *mm* and −1 to the off-diagonal entries *nm* and *mn* of the matrix **A**. In essence, the suggested assembly procedure replicates the assembly procedure commonly used in the finite element method.^47^

In this work, computer models with up to *N* ≈ 3.2 · 10^4^ beads are studied, and it takes about 90 s on an Apple M3 Max processor to calculate the eigenvalues λ_*p*_ of the connectivity matrix **A** of such a network using the eigenvalue command of Mathematica.

### Molecular Dynamics Simulations

The coarse-grained Kremer-Grest molecular dynamics (MD) simulations are used.^48,49^ Periodic end-linked network models are generated using a collision-diffusion procedure starting from equilibrated melts of identical difunctional precursor chains and tetra-functional cross-linkers.^44,45,50^ The extent of reaction *p* is defined as the fraction of reacted sites of the cross-linkers. The stoichiometric ratio *r* is defined as the initial ratio of the reactive sites of the cross-linkers and the precursor chains. Network models with 100 identical strands with *K* internal Lennard-Jones beads connected by finite extensible nonlinear elastic (FENE) bonds are studied. The results are averaged over 25 different realizations. After end-linking, equilibration runs of 4 · 10^8^ MD steps are carried out followed by production runs of 4 · 10^8^ MD steps (ca. 20 μs) in which the shear stress autocorrelation function *C*(*t*) is estimated using the on the fly multiple-tau correlator^9,51−53^ as follows [cf. eqs 11 and 12]:^54,55^
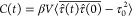
25where β = 1/*k*_B_*T* is the inverse temperature, τ̂(t) is the instantaneous shear stress at time *t*, the overbar denotes the averaging over all possible time origins of the MD trajectory of a particular realization of a given network, the angular brackets are for the averaging over 25 realizations, and τ_0_ is the residual, frozen stress that builds up upon curing a realization in a small periodic cubic box without letting the stress to relax by allowing for a triclinic box, which is evaluated as the mean stress over the whole sampling trajectory. For more computational details, see the Appendix.

**Figure 3:**
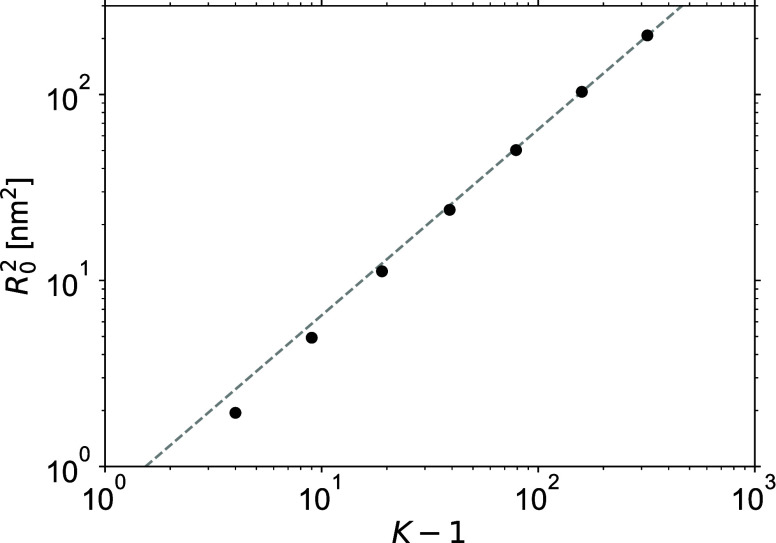
Conversion to the Kuhn representation. The symbols are MD estimates of the mean square end-to-end distance *R*_0_^2^ of the chains, and the dashed line is a linear fit with the zero intersect to the estimates.

The conversion to the Kuhn representation is achieved by replacing each network strand with (*K*+1) FENE bonds by a Gaussian strand with a nearest-integer of 0.584(*K*+1) Kuhn segments. The conversion factor 0.584 is obtained from MD simulations with precursor melts, see Figure 3. One can see that for chains with *K* = 19 beads and longer, the mean square end-to-end distance scales linearly with the number of FENE bonds in the chains while for the shorter chains the linear dependence does not hold well. In this work, network models with strands consisting from *K* = 18 to 318 beads are studied, corresponding to molar weights from *M*_n_ = 2900 g/mol to 51000 g/mol, which is a representative range for real end-linked polydimethylsiloxane (PDMS) networks.

Using the linear fit of Figure 3 and the mean FENE bond length 0.594 nm from the MD simulations, the parameters of the Kuhn representation are obtained, the segment length *b*_K_ = 1.09 nm and molar mass *M*_K_ = 296 g/mol. As a validation, the linear fit corresponds to 0.0404 nm^2^/(g/mol), which agrees well with 0.0422 nm^2^/(g/mol) measured for PDMS by small-angle neutron scattering.^56,57^

### Perfect Networks

**Figure 4:**
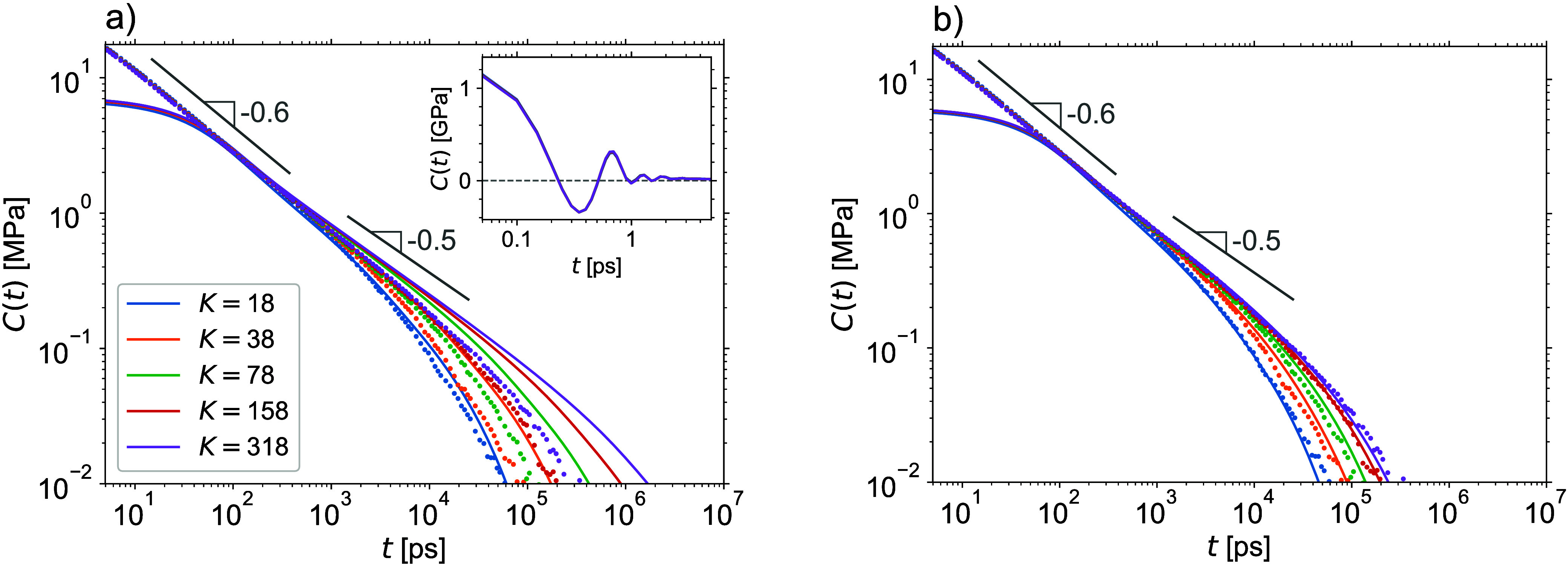
Stress autocorrelation functions of perfect networks. The symbols are multiple-tau correlator MD estimates, while the lines are theoretical predictions, both shown beginning from 5 ps, which corresponds to 100 MD steps. (a) Comparison with the model of unentangled dynamics. In the inset, short-time damped-oscillatory bond-vibration MD dynamics is shown by lines connecting the multiple-tau correlator data points. (b) Comparison with the model of entangled dynamics. In both cases, the same mapping factors for time and stress are used.

Figure 4a compares MD estimates of the autocorrelation functions *C*(*t*) of nearly perfect, fully cured stoichimetric end-linked polymer networks (*p* = 1 and *r* = 1, termed perfect for brevity, even though they involve some loops as will be discussed below) with the theoretical predictions of eqs 7 and 12 obtained using the same MD network realizations but converted to the Kuhn representation. For the theoretical predictions, the model of unentangled dynamics is first considered, see Figure 1. The mapping between the two sets of results is achieved by horizontal and vertical shifts of the theoretical predictions in order to superimpose them with the MD predictions at a time of 100 ps, where the symbols and the lines of Figure 4a collapse all together. The horizontal mapping is physically required to fix the intrinsic Langevin time ζ/*k*, which is a free parameter of the theory, whereas the vertical mapping is a numerical correction reflecting the approximate nature of both coarse-grained Kremer-Grest MD simulations and the conversion procedure to the Kuhn representation. The resulting mapping factors are ζ/*k* = 432 ps for time and 0.757 for stress, with the latter one being just a small correction on the logarithmic scale.

The inset of Figure 4a shows short-time MD dynamics, which is independent of *K* and is related to damped vibrations of FENE bonds. The *C*(*t*) begin from *C*(0) ≈ 1.7 GPa, which is representative of the high-frequency shear modulus of glassy polymers but is much larger than the equilibrium shear modulus of polymer networks, which is commonly below 1 MPa. This damped-oscillatory regime is independent of the state of the system and is similarly observed in melt simulations with the precursor chains (not shown here). At such short times the system does simply not realize whether it is in a liquid (melt) or solid (network) state. Because of the coarse-grained nature of the KG MD simulations, this short-time damped-oscillatory regime is obviously artificial, it is not representative of the underlying atomistic dynamics of chemical PDMS repeat units, and we shall not further elaborate on it. No such damped-oscillatory modes of motion exist in the theoretical Kuhn-scale description.

Figure 4 shows that at times *t* < 100 ps all theoretical *C*(*t*) curves merge together and level off, meaning that time *t* becomes smaller than the shortest Rouse time τ_*N*–1_, see eqs 7 and 21. At times *t* > 5 ps, the MD results suggest a power law dynamics *C*(*t*) ∝ *t*^–0.6^ followed by an exponential cutoff. This power-law scaling behavior was already studied and discussed by Likhtman et al. in their paper on MD simulations of polymer melts.^9^ They performed additional simulations with modified force field parameters and attributed this scaling behavior to the colloidal or glassy modes of stress relaxation. In polymer melts, this power-law regime is terminated by the chain reptation while in the perfect polymer networks it is directly terminated by the terminal exponential relaxation. The theoretical predictions replicate this power-law behavior at times between ca. 50 and 1000 ps but then for the networks with longer strands they follow the characteristic Rouse behavior *C*(*t*) ∝ *t*^–0.5^, see eq 23. As a consequence, it is only for the network with *K* = 18 that the predictions obtained with the model of unentangled dynamics match the MD results, which is not surprising given that for PDMS precursor melts the entanglement chain length is *K*_e_ ≈ 70 beads.^58^

In the KG MD simulations, the strands do not cross each other and themselves so that the presence of entanglements is taken into account automatically. In our model of entangled dynamics, the entanglements are introduced as additional entropic springs connecting adjacent beads of network strands, see Figure 1. The entanglement number density ϵ is defined based on the Miller-Macosko theory (MMT) formula for the equilibrium shear modulus *G*_eq_:^43^
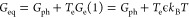
26where *G*_ph_ is the phantom, cross-link contribution to *G*_eq_, *G*_e_(1) is the entanglement modulus of a fully polymerized stoichiometric network, and *T*_e_ is the Langley’s trapping factor,^14,59−61^ the probability that all four chain ends coming from an entanglement lead into the infinite network, see eqs 37–41. For the perfect, fully polymerized stoichiometric networks the trapping factor *T*_e_ = 1. Following on from our recent studies on the validity of the MMT for end-linked polymer networks formed from bulk,^44,45^ we have derived ϵ = 0.0570 nm^–3^ from eq 26 using a value of *G*_e_(1)=0.236 MPa obtained for end-linked PDMS networks by fitting the MMT predictions to stress-relaxation MD estimates of *G*_eq_.^44^

For a network realization of volume *V*, the last frame of the sampling MD trajectory is used to randomly draw a set of *ϵV* pairs of adjacent internal strands’ beads separated by less than 2σ, where σ = 0.616 nm is the Lennard-Jones unit of length, see the Appendix, and the beads in the pairs are linked together. The network is converted to the Kuhn representation by treating these pairs of linked beads as pairs of trifunctional network junctions connected by an entropic spring with stiffness coefficient *k*, the connectivity matrix **A** is assembled, and its eigenvalues λ_*p*_ are used to calculate the stress autocorrelation function *C*(*t*) using eqs 7 and 12.

Figure 4b shows that for times *t* < 10^3^ ps, there is no difference between theoretical predictions obtained with the models of entangled and unentangled network dynamics. On these times, the molecular motions are localized and they are not influenced by the entanglements. However, on longer times the entangled dynamics becomes faster than the unentangled one, it approximately scales as *C*(*t*) ∝ *t*^–0.6^, and the theoretical and MD stress autocorrelation functions agree well. We explain this by the fact that in the perfect networks, there are no soluble and dangling molecular structures so the beads and the entanglements can only execute localized vibrational motions around their mean positions and hence, the underlying assumptions of the model of entangled dynamics are largely satisfied.

### Imperfect Networks

The proposed molecular model automatically takes into account various topological defects commonly occurring in real polymer networks, see Figure 1. To further test the model, we consider imperfect, nonstoichiometric end-linked polymer networks with *r* = 2 (excess of cross-linkers) and *p* = 0.5 (all reactive end-groups of the difunctional precursor chains reacted). Using MD simulations as described above for the perfect networks, network realizations consisting of 100 tetra-functional cross-linkers linked by 100 strands with *K* = 18 and 158 internal beads are generated and their stress autocorrelation functions are estimated by averaging over 25 realizations.

Table 1 compares topological parameters of the perfect and imperfect networks as determined by the force-relaxation procedure.^44,45,62,63^ One can see that the fractions of the primary and secondary loops are relatively small, which is common to polymer networks formed from bulk, in contrast to those formed from solution where the loop fractions can be significantly larger.^64−66^ In the perfect networks there are no soluble and dangling structures while in the imperfect networks about 40% of their strands are involved in soluble and dangling structures. As a consequence, the equilibrium shear modulus *G*_eq_ of the imperfect networks is smaller than that of the perfect networks.

**Table 1:** Summary of Topological Parameters of Perfect and Imperfect Networks^a^

	*K*	*M*_n_ [g/mol]	*w*_sol_	*w*_dang_	*w*_1_	*w*_2_	*T*_e_	*G*_eq_ [MPa]
perfect	18	2900	0	0	0.048	0.045	1	0.639
	38	6120	0	0	0.035	0.051	1	0.429
	78	12560	0	0	0.027	0.048	1	0.330
	158	25450	0	0	0.024	0.050	1	0.282
	318	51200	0	0	0.019	0.059	1	0.258
imperfect	18	2900	0.073	0.377	0.033	0.029	0.341	0.151
	158	25450	0.070	0.306	0.015	0.020	0.341	0.089

a The *w*_sol_ and *w*_dang_ are the fractions of the network strands participating in the soluble and dangling structures, while *w*_1_ and *w*_2_ in the primary and secondary loops, respectively, and *T*_e_ and *G*_eq_ are the MMT predictions for the trapping factor and the equilibrium shear modulus obtained with eqs 38–41, see the Appendix.

**Figure 5:**
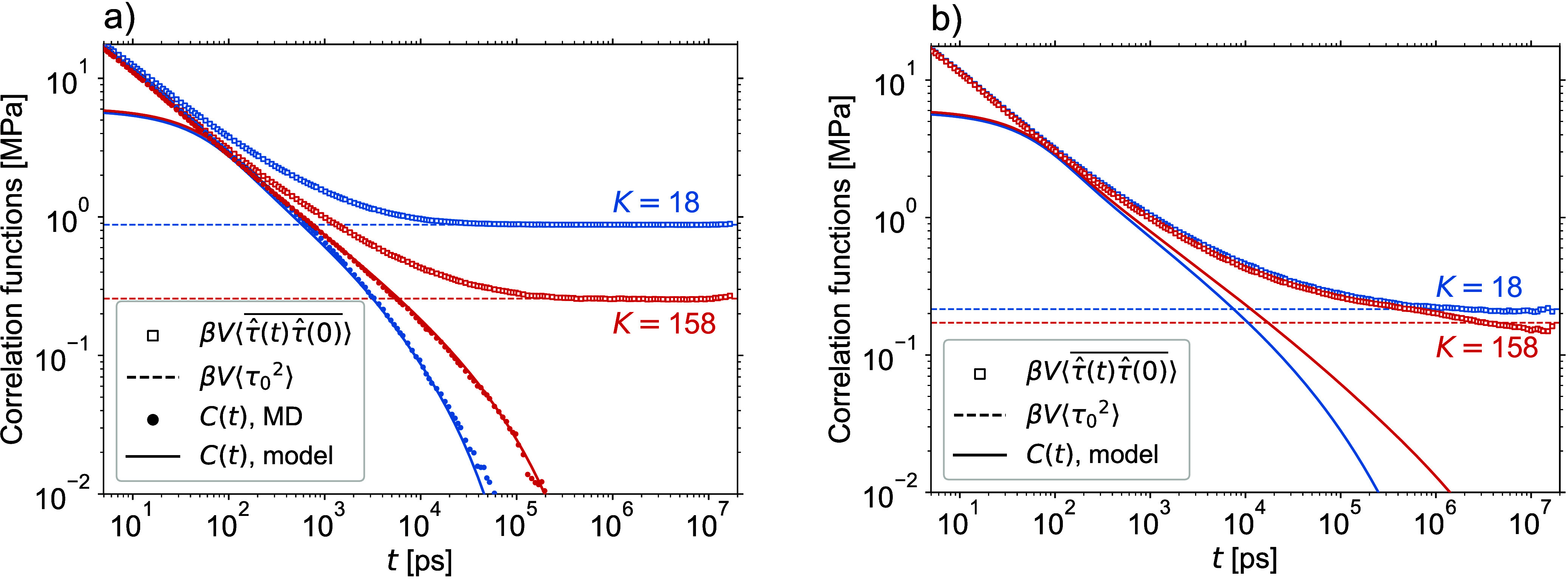
Residual-stress contribution. Open and filled circles are, respectively, the direct MD estimates and derived predictions for *C*(*t*). The dashed lines show the residual-stress contributions calculated using the mean stresses evaluated over the whole sampling MD trajectories. The solid lines are the theoretical predictions. (a) Perfect networks. (b) Imperfect networks.

Figure 5 shows the time correlation functions of total instantaneous stress τ̂(*t*) of the perfect and imperfect networks, which are directly accessible in MD simulations. Evidently, they converge to nonzero plateau values as *t* → *∞*. To obtain the required stress autocorrelation function *C*(*t*), which must decay to 0 as *t* → *∞*, the parasite contribution of residual stress τ_0_ must be subtracted, see eq 25. Figure 5a shows that for the perfect networks, the plateau values are reliably reached during the simulations and the subtraction can accurately be performed. Notice that a high accuracy in τ_0_ is required to reliably evaluate the long-time tail of *C*(*t*), which encodes the practically relevant viscoelastic storage and loss moduli.

Physically, the Langley’s trapping factor *T*_e_ gives the fraction of the trapped entanglements, those that connect elastically effective strands of the network, see eqs 26 and 41. Such strands can be identified by the force-relaxation procedure.^44,45,62^ Following on from the successful validation of MMT predictions for *G*_eq_,^43−45^ upon assembly of the connectivity matrix **A** of imperfect networks, we only consider the trapped entanglements but disregard the temporary entanglements, which connect to at least one elastically ineffective strand, see Figure 1. The resulting fractions of trapped entanglements are 0.387 ± 0.145 for the imperfect network with *K* = 18 and 0.327 ± 0.091 for *K* = 158, which is close to the MMT trapping factor *T*_e_ = 0.341 used to predict *G*_eq_, see Table 1 and eqs 26 and 37–41.

The predicted *C*(*t*) functions of imperfect networks are shown in Figure 5b. One can see that at longer times they exhibit additional relaxation modes as compared to the *C*(*t*) of perfect networks shown in Figure 5a, which is due to the slowly relaxing soluble and dangling structures, see Table 1. Since for the imperfect networks the MD simulations are evidently not long enough to reach the plateau regime, no reliable subtraction of the residual-stress contribution can be done and so only theoretically predicted *C*(*t*) are shown in Figure 5b.

The suggested simple model for the dynamics of entangled polymer networks evidently precludes the reptation movement of free polymer chains through polymer networks as well as it does not nearly follow the complicated kinematics of pull-out and re-entry mechanisms of thermal relaxation of dangling chains and their branched molecular structures. However, the PDMS precursor chains commonly used to produce real end-linked polymer networks are relatively short, with molar masses from 10^3^ to 10^5^ g/mol, while the entanglement molar mass in high-molar mass PDMS melts is *M*_e_ ≈ 1.2 × 10^4^ g/mol.^57^ As a result, there are at most several entanglements per network strand, and we surmise that the proposed model of entangled network dynamics may prove helpful for practical trend studies of linear viscoelastic properties of polymer networks.

In order to further understand the scope of validity of the proposed model, we shall compare its predicted viscoelastic storage and loss moduli with the MD estimates.

### Viscoelastic moduli

**Figure 6:**
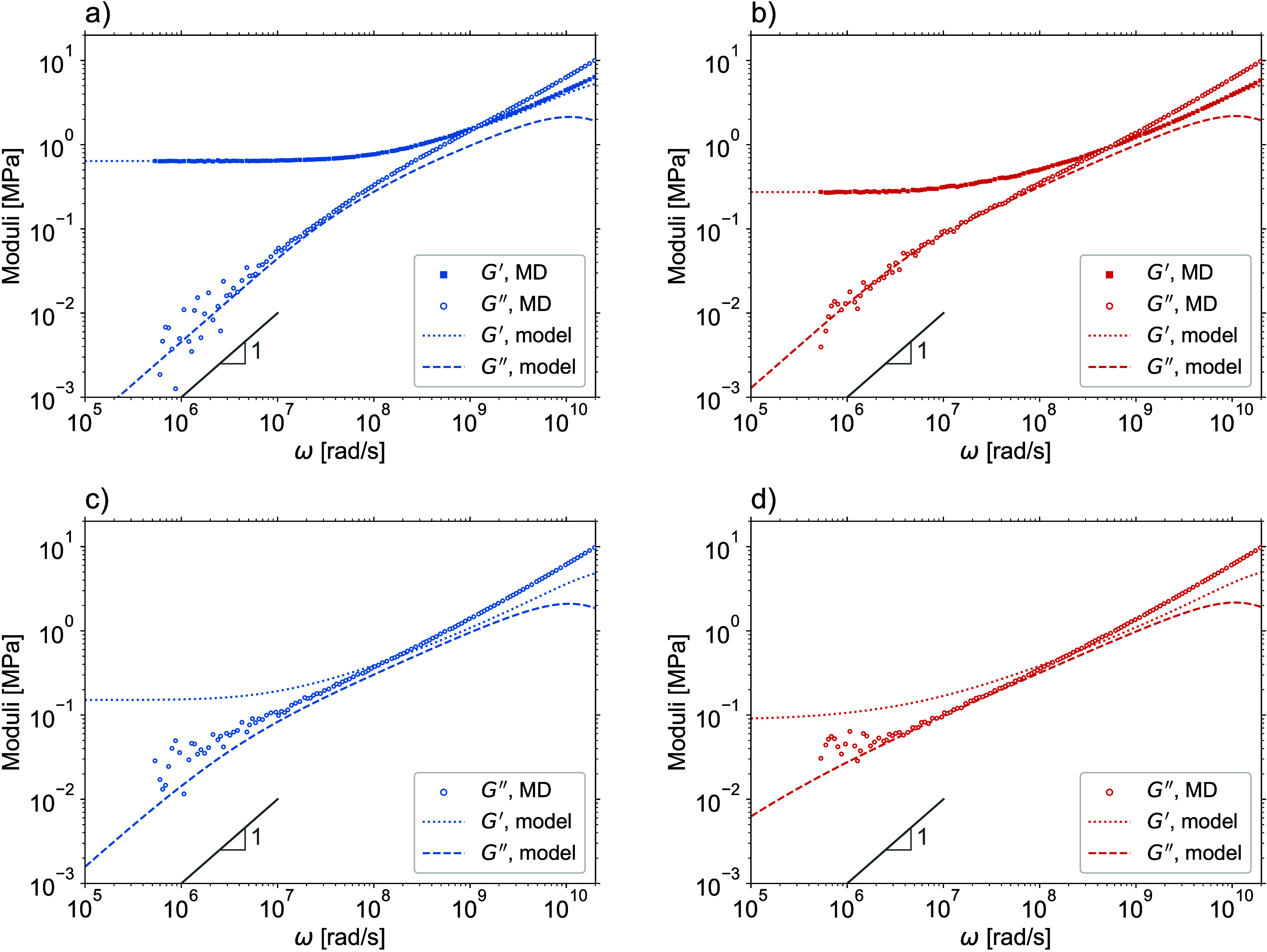
Viscoelastic moduli. The lines are theoretical predictions and the symbols are MD estimates. The values of equilibrium modulus *G*_eq_ are taken from Table 1. The bars with slope 1 are for the terminal exponential regime of *G*″. (a) Perfect network with *N* = 18. (b) Perfect network with *N* = 158. (c) Imperfect network with *N* = 18. (d) Imperfect network with *N* = 158. Notice that at high frequencies, the theoretical *G*″ curves deviate from the MD ones, which is due to the high frequency limit of the Rouse description.

On the basis of eqs 7, 12, and 13, the viscoelastic storage and loss moduli, *G*′(ω) and *G*″(ω), respectively, are given by the following expressions:
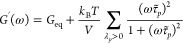
27
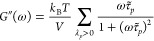
28where ω is the frequency, , and the sum is taken over all vibrational normal modes. The model predictions for the viscoelastic moduli of perfect and imperfect networks of Table 1 are shown by lines in Figure 6.

In MD simulations, the complex shear modulus G*(ω) is evaluated based on the multiple-tau correlator estimates *C*_*k*_ of *C*(*t*) collected at times *t*_*k*_ (with *k* = 0, 1, ..., *M* and *t*_0_ = 0) using the direct transform method,^67^ see eqs 35 and 36. By separating G*(ω) of eq 36 into real and imaginary parts, one obtains
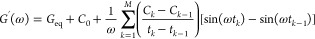
29
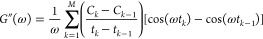
30Notice that the residual stress τ_0_ of eq 25 cancels out in every term of the sums. Therefore, the *G*″ can be evaluated even from the simulations in which the τ_0_ has not yet fully converged, as those with the imperfect networks shown in Figure 5b. However, the τ_0_ contributes to the *G*′ via the term *C*_0_ so accurate estimates of τ_0_ are required for reliable evaluation of *G*′, which are available for the perfect networks but not for the imperfect networks, see Figure 5.

Figures 6a and 6b show that for frequencies ω < 10^8^ rad/s, the theoretical predictions and MD estimates of *G*′ and *G*″ of the perfect networks agree well and both moduli enter into the terminal exponential relaxation regime with *G*′ → *G*_eq_ and *G*″ ∼ ω. However, because of the poor statistics in the long-time tails of *C*(*t*), the MD estimates become noisy and less reliable in the low-frequency range ω < 10^6^ rad/s.

Figures 6c and 6d show that the theoretical predictions and MD estimates for the *G*″ of the imperfect networks also agree well, even though the MD estimates have not yet entered into the terminal exponential regime. For this, longer MD runs are needed, which is impractical today. On the contrary, the theoretical predictions for *G*′ and *G*″ are available at any frequency, with the terminal relaxation time being determined by the smallest nonzero eigenvalue λ_*p*_ of the connectivity matrix **A**. However, despite the favorable validation for the overlapping frequency range, one should emphasize once more that the suggested model for entangled network dynamics is possibly oversimplified so the model predictions may not necessarily be quantitative for the low frequencies ω < 10^6^ rad/s, where the reliable MD estimates are not available today. Nonetheless, it will be interesting to see if and how this simple proposed model can be used for practical predictions of the trends caused by molecular modifications of polymer networks.

Interestingly, even though the fractions of soluble and dangling strands in the imperfect networks are relatively small (cf. Table 1), they nevertheless significantly enhance the loss modulus *G*″ at low frequencies, compare Figures 6a and 6b with Figures 6c and 6d, respectively. This enhancement should already be anticipated from Figure 5, where the *C*(*t*) curves of the imperfect networks noticeably deviate from the power law behavior exhibited by the *C*(*t*) curves of the perfect networks.

For a single network realization with *K* = 158 beads, about 10^7^ single-core CPU seconds on the mainframe Euler cluster of ETH Zürich (https://scicomp.ethz.ch/wiki/Euler) are required for the equlibration and sampling MD runs using LAMMPS^68^ while it takes only about 10 s on Apple M3 Max processor to obtain the corresponding theoretical predictions using a Mathematica script, so a speed up of 10^6^ times. However, even with such massive parallel supercomputing simulations, the MD estimates are still limited to a high frequency range of ω > 10^6^ rad/s while the theoretical predictions cover the whole range of frequencies ω < 10^8^ rad/s. And our theoretical calculations can be significantly accelerated by running the existing highly optimized eigenvalue solvers on the fast mainframe computers, thus allowing for efficient design studies with large representative models of polymer networks.

## Conclusions

Using the general form of the linearized Langevin equation, a molecular Kuhn-scale model was presented for the stress relaxation dynamics of entangled polymer networks. Based on the fluctuation–dissipation theorem, the stress relaxation modulus was derived using the normal mode representation. The entanglements were introduced as additional entropic springs connecting internal beads of network strands. The model was validated by comparing predicted stress relaxation and viscoelastic storage and loss moduli with the estimates from molecular dynamics simulations, obtained using the same computer models of both perfect and imperfect end-linked polymer networks. It was shown that for the overlapping ranges of times and frequencies, the model predictions and MD estimates agree well.

In the perfect networks, there are no soluble and dangling molecular structures so the beads and the (trapped) entanglements execute localized vibrational motions around their mean positions and the model assumptions are largely satisfied. However, for the imperfect networks with different soluble and dangling molecular structures, the suggested molecular model of entangled network dynamics is possibly oversimplified and further studies are needed to better understand its scope of practical validity and to suggest possible refinements.

In this work, relatively small computer network models with 100 strands have been studied. However, for practically relevant predictions larger models should be used, with more representative spectra of branched soluble and dangling structures and loops. Such larger models can be generated using the Monte Carlo (MC) procedure introduced and used in previous works.^62,63,69,70^ And we shall use such MC models in our future work to compare our theoretical predictions with experimental data and to study the relationships between the viscoelastic moduli and the kinematics of underlying thermal motion of different molecular fragments including soluble and dangling structures and loops, by analyzing the eigenvalues and corresponding eigenvectors of the connectivity matrix **A**.

## Appendix

In the coarse-grained Kremer-Grest molecular dynamics simulations, a polymer chain is represented as a sequence of beads connected by springs.^48,49^ All beads interact via a repulsive, truncated and shifted to zero at the minimum Lennard-Jones (LJ) potential

31

where *r* is the distance between the beads and σ and ϵ are the LJ units of length and energy, respectively. The unit of time is τ = σ(*m*/ϵ)^1/2^, where *m* is the bead mass. Additionally, chemically bonded beads interact via the finite extensible nonlinear elastic (FENE) potential

32

where the maximum extension *r*_0_ = 1.5σ and the spring constant κ = 30ϵ/σ^2^ were adjusted so that the chains do not cross each other. A bead density of 0.85σ^–3^ is used in the simulations.

The MD simulations are carried out in the *NVT* ensemble using a velocity-Verlet integrator with a time step of *δt* = 0.01τ using LAMMPS.^68^ The simulations are performed assuming unit LJ scales of length, energy and mass. A Langevin thermostat with a damping constant of 0.02/*δt* is employed. It was shown by linking experimental and simulation results for polydimethylsiloxane (PDMS) melts at *T* = 298 K that the basis conversion factors from the LJ to SI units are 1ϵ = 4.11 · 10^–21^ J for energy, 1σ = 0.616 nm for distance, 1*m* = 2.67 · 10^–25^ kg for mass (corresponding to 161 g/mol, which is about two chemical repeat units of PDMS),^58^ resulting in the derived conversion factors 4.97 ps for time and ϵ/σ^3^ = 17.6 MPa for pressure.

The shear stress autocorrelation function is estimated using the expression:^9,53^

33

where  and  are, respectively, the shear and normal components of the instantaneous virial stress tensor **σ̂**(t), with indexes α ≠ β assuming *x*, *y* and *z*. This expression is obtained by averaging the in-plane shear stress over all possible differently oriented planes. The related expression for the mean (residual) stress is as follows:

34

Using the causality principle, the complex modulus *G**(ω) can be calculated as follows:

35

where *G*′(ω) and *G*″(ω) are the loss and storage moduli, respectively. In MD simulations, the stress autocorrelation function *C*(*t*) is evaluated using the multiple-tau correlator and is sampled at a finite set of data points (*t*_*k*_,*C*_*k*_), where *k* = 0, 1, ..., *M* and *t*_0_ = 0. By interpolating this data set with a piecewise linear function and assuming that *Ċ*(*t*) = 0 for *t* > *t*_*M*_ one finds

36

eq 36 constitute the basis for a direct numerical transform of the stress autocorrelation function *C*(*t*) into the storage and loss moduli without using any preconceived model. For polymer melts, this method was suggested by Evans et al.^67^ In this work, an in-house complex-arithmetic Mathematica script is used to evaluate the complex modulus *G**(ω) using as input the MD (*t*_*k*_,*C*_*k*_) data points obtained with the multiple-tau correlator and the MMT estimates of the equilibrium shear modulus *G*_eq_.

The Miller-Macosko theory (MMT) assumes that the contributions of the cross-links, *G*_ph_, and the entanglements, *G*_e_, to the equilibrium shear modulus *G*_eq_ are additive:^43^

37

For the end-linked polymer networks obtained by stepwise polymerization of difunctional precursor chains B_2_ and tetra-functional cross-linkers A_*f*_ (in this study *f* = 4), the MMT provides the following analytical formula for *G*_ph_:

38

where  is the initial number density of the cross-linkers and

39

is the probability that in the cured network a cross-link chosen at random is an effective network junction *X*_*m*,*f*_ of degree *m*, the front factor on the right-hand side is the binomial coefficient, and for the studied networks with *f* = 4

40

which is the probability of the event that looking out from a randomly chosen reactive site of a network junction leads to a finite chain rather than to the infinite network.

The trapping factor *T*_e_, the probability that all four chain ends coming from an entanglement lead into the network, is given by

41

where *P*(*F*_*B*_^out^) is the probability of the event that looking out of from one of the two ends of a strand chosen at random leads to a finite chain rather than to the infinite network.
